# A semantic feature enhanced YOLOv5-based network for polyp detection from colonoscopy images

**DOI:** 10.1038/s41598-024-66642-5

**Published:** 2024-07-05

**Authors:** Jing-Jing Wan, Peng-Cheng Zhu, Bo-Lun Chen, Yong-Tao Yu

**Affiliations:** 1 Department of Gastroenterology, The Second People’s Hospital of Huai’an, The Affiliated Huai’an Hospital of Xuzhou Medical University, Huaian, 223023 Jiangsu China; 2 Faculty of Computer and Software Engineering, Huaiyin Institute of Technology, Huaian, 223003 China

**Keywords:** Image processing, Machine learning

## Abstract

Colorectal cancer (CRC) is a common digestive system tumor with high morbidity and mortality worldwide. At present, the use of computer-assisted colonoscopy technology to detect polyps is relatively mature, but it still faces some challenges, such as missed or false detection of polyps. Therefore, how to improve the detection rate of polyps more accurately is the key to colonoscopy. To solve this problem, this paper proposes an improved YOLOv5-based cancer polyp detection method for colorectal cancer. The method is designed with a new structure called P-C3 incorporated into the backbone and neck network of the model to enhance the expression of features. In addition, a contextual feature augmentation module was introduced to the bottom of the backbone network to increase the receptive field for multi-scale feature information and to focus on polyp features by coordinate attention mechanism. The experimental results show that compared with some traditional target detection algorithms, the model proposed in this paper has significant advantages for the detection accuracy of polyp, especially in the recall rate, which largely solves the problem of missed detection of polyps. This study will contribute to improve the polyp/adenoma detection rate of endoscopists in the process of colonoscopy, and also has important significance for the development of clinical work.

## Introduction

Colorectal cancer (CRC) is a malignant tumor of the digestive system that usually occurs in the colon or rectum. It is one of the most common tumors in the world and one of the leading causes of death. According to the World Health Organization, CRC is the third most common cancer in the world, after lung and breast cancer. According to statistics, about 2 million people around the world are diagnosed with CRC every year, and about 800,000 of them die from the disease, which has a great impact on people’s physical and mental health and quality of life^1^. The vast majority of CRCs evolve gradually from colorectal polyps^2^. Data show that early detection and removal of polyps is important to prevent the development of CRC and improve cure and survival rates, reducing CRC-related mortality by 70%^3^. Therefore, early detection and identification of polyp types is crucial to preventing CRC and changing the outcome^4–8^.

Colonoscopy is considered the most direct and effective way to detect colorectal cancer, and the prevention of colorectal cancer by colonoscopy benefits from the improvement of adenoma detection rate (ADR). Extensive data show that for every 1.0% increase in adenoma detection by colonoscopy, the incidence of interstage CRC decreases by 3.0–6.0%. It is evident that there is a significant negative correlation between ADR and CRC. In addition, historical data show that there is a good correlation between polyp detection rate (PDR) and ADR, therefore, PDR can be used as a proxy for ADR for colonoscopy quality^9^. However, due to individual differences in the skill level of endoscopists, the detection rate of adenomas varies from 7 to 53%. The detection and timely removal of adenomatous polyps by colonoscopy is effective in reducing the morbidity and mortality of CRC, and reports have shown that the mortality of CRC can be reduced by more than 50%^10,11^. However, polyps may be missed due to differences in lighting conditions of endoscopes, texture, appearance, and overlapping morphology among polyps, as well as individual differences in the skill level of endoscopists, and according to incomplete statistics, the polyp detection rate is only 78%, making a significant portion of polyps missed^12^. Therefore, seeking other means to increase the polyp detection rate of colonoscopy and reduce the number of polyps missed, thus reducing the incidence of CRC has become a top priority in current research^13^.

In recent years, with the continuous development of information technology, it has become a trend to utilize computers for assistance in colonoscopy. Computer-aided colonoscopy (CACC) is a new examination method that combines computer technology and endoscopic technology, aiming to improve the diagnostic accuracy and detection rate of colon cancer. Currently, with the continuous maturation of artificial intelligence (AI), the use of AI-assisted colon cancer examination has become one of the hotspots of current research. Barua et al. conducted a systematic search for the application of AI in colonoscopy detection, including relevant literature in Medline, Embase, and Cochrane Central databases. Their effectiveness in detecting polyps, adenomas, and colorectal cancer was evaluated by comparing the relative risk, absolute risk, and mean difference between colonoscopies assisted with AI and those not using AI. The results of the evaluation showed that the AI-based polyp detection system was effective in improving the detection of non-advanced adenomas and smaller polyps during colonoscopy^14^.

Although AI has achieved good results in assisting digestive endoscopy detection, there are still many problems worth exploring. For example, from the feature level: the limitations of colonoscopy equipment lead to the phenomenon of blurred edges between adjacent tissues in colorectal imaging, as well as the problems of low contrast, insufficient light, and noise interference in the polyp image itself, which makes the performance of traditional detection algorithms in the process of extracting polyp features unsatisfactory. better features cannot be obtained. From the perspective of algorithm performance: Because the traditional classification algorithm has a high accuracy in detecting benign polyps, but the detection accuracy for malignant polyps is not high, and false negatives (misdiagnosis) may occur. In addition, the time complexity of some target detection algorithms is high, which is difficult to meet the demand of real-time analysis of medical images^15^.

Therefore, how to effectively extract the characteristics of polyps and design a high-precision, low-complexity classification method has been a thorny problem for researchers. This paper combines the characteristics of polyp image data and uses the advantages of real-time and accuracy of YOLO algorithm to design the algorithm. The main contributions are as follows: (1) A new type of module named P-C3 is designed using the P-BottleNeck structure to improve polyp detection accuracy in model. (2) Aiming at the problem that polyps in colonoscopy images are small and difficult to be detected, a contextual feature fusion module is designed to enhance the contextual feature information of tiny polyps. (3) Adding a coordinated attention mechanism to improve the high-quality extraction of polyp features in colorectal images by the network model. (4) Taking into account the accuracy and speed of the model, YOLOv5 was used as the baseline for the polyp detection model, and the WCYZ polyp dataset was produced which is expanded using Mosaic to validate the superiority of the method in this paper.

## Related works

At present, target detection algorithms are mainly divided into three categories: traditional target detection algorithms, two-stage-based detection models, and single-stage-based detection models. Among them, the traditional detection model mainly includes three components: region selection, feature extraction, and classifier classification. The two-stage detection model mainly generates candidate regions, and then classifies and predicts the location coordinates of the candidate regions. The single-stage detection model is a kind of method that uses an end-to-end fully convolutional neural network (CNN) to complete the input from the original image to the output object category and location.

### Traditional polyp detection model

As is known to all, the analysis of endoscopic images is mainly performed by endoscopists, which leads to the fact that the accuracy of the judgment of endoscopic images is very dependent on the level of the physician. However, accurate analysis of endoscopic images is difficult for inexperienced endoscopists. The development of a real-time system capable of automatic polyp detection could help physicians improve their ability to analyze endoscopic images. Computer-aided diagnosis (CAD) of colonoscopy has always been a hot spot in artificial intelligence research. CAD can display in real time and prompt endoscopists to pay attention to polyps that may be overlooked, thereby improving the detection rate of adenoma^16,17^.

Traditional polyp detection algorithms usually use artificial features such as texture, shape, and color features to detect polyps. Krishnan et al. devised a method to obtain the location of polyps in images by extracting haustra folds in medical images^18^. Kang et al. designed a fast detection system considering the real-time nature of polyp detection in practical applications. The system segments medical images and then categorizes them separately to find out the location of polyps^19^. Bernal et al. used valley information to design a detection algorithm to determine intact polyp boundaries based on the properties of the polyp surface^20^. Alexandre et al. devised a method to binary classify each segmented polyp sub-image using support vector machine (SVM) to determine whether or not it contains polyps^21^. Li et al. proposed a method that uses multiple patches of different sizes to represent polyp images, each of which is classified by SVM to determine if it is a polyp^22^. Manouchehri et al. designed a new network model for frame detection of polyps based on Visual Geometry Group Net (VGGNet), based on which polyps were segmented using post-processing methods in a fully convolutional neural network^23^. Mostafiz et al. performed feature extraction for the color of polyps, which in turn led to the design of an intelligent system for gastrointestinal polyp detection in endoscopic videos by fusing 2D empirical modal decomposition and deep neural network features^24^. Billah et al. designed a new system to address the shortcomings of the traditional algorithm with a high leakage rate, which automatically extracts the colored wavelet features in the video frames for training the linear SVM to achieve the classification of polyps^25^. Hasan et al. designed a method for gastrointestinal polyp detection by fusing contour wavelet transform and neural features. The method uses minimum redundancy maximum relevance (MRMR) dimensionality reduction method to extract deep features from the image and then SVM to diagnose gastrointestinal polyps and label the detected polyp regions^26^.

Traditional polyp detection models usually adopt a strategy based on sliding windows in region selection, but the sliding windows are of different sizes and are not targeted, resulting in redundant windows and high time complexity. In addition, the robustness of the feature extraction method is poor due to polyp image morphological diversity, illumination change diversity and background diversity. For example, due to the smooth surface of some polyps, they show the same shape-texture features as the normal lining in endoscopic images, and traditional algorithms tend to miss such polyps; the normal inner wall of the colon has a raised structure, and traditional algorithms are easy to misdetect it as polyps. Therefore, traditional polyp detection algorithms are difficult to complete the detection task well. 

### Two-stage polyp detection model

The last several years, with the excellent performance of AlexNet on ImageNet, deep learning has shined in the field of computer vision, and various performances far exceed traditional algorithms, and the field of polyp detection is no exception. To find the optimal polyp detection algorithm, Bernal et al. investigated a large number of deep learning detection methods as well as the original manual feature extraction-based detection methods. A comparison of the study reveals that the detection methods using the deep learning approach show superior performance for both single image detection and medical image frame detection, and the deep learning approach is more convenient than the manual feature extraction approach in practical clinical applications^27^.

In the design of the two-stage detection model, CNN have been applied to practical clinical polyp detection, and the leakage rate has been kept within a low range. Tajbakhsh et al. proposed a computer-aided detection-based hybrid context-shape polyp detection method. The algorithm first removes the non-polyp edges from the edge map using contextual information and then locates the polyp candidate regions in the improved edge map using multi-scale temporal information based on color and texture^28^. In the detection of colon cancer polyps, the convolutional neural network model is susceptible to small perturbations and noise, which can miss the detection of neighboring polyps in medical image frames, increasing the number of false negatives and a decrease in the accuracy of the detection model. Qadir et al. designed a two-stage approach using region-of-interest and false-negative reduction units, respectively. The algorithm provides an overall performance improvement in terms of sensitivity, accuracy and specificity^29^.

Mo et al. studied and compared many colonoscopic polyp detection methods and found that: traditional methods are difficult to be applied to practical applications due to lower accuracy and higher time complexity, while neural network-based detection models are extraordinary in performance, among which the Faster R-CNN algorithm polyp detection based on Faster R-CNN algorithms performs the best and achieves satisfactory results, which can be used in clinical practice^30^. Qadir et al. analyzed the differences in polyps in terms of contrast, size, and texture, used Mask R-CNN as a baseline model and replaced its feature extractors, and compared the improvement in detection and segmentation performance of each feature extractor. Finally, an integrated method is proposed for polyp detection and segmentation with good results on the MICCAI dataset^31^. Tashk et al. modified the region suggestion network to localize polyps from video frames acquired from a colon capsule endoscope. This method can accurately detect polyps in the video stream and, in addition to that, provide a predictive score for the risk of them being malignant tumors^32^. Patel et al. used several neural networks to classify polyps and compared their effectiveness on a publicly available polyp dataset. Ultimately, the VGG-19 model was found to perform better on the dataset than that of residual network (ResNet), Densely Connected Convolutional Network (DenseNets) and Squeeze-and-Excitation Networks (SENet) and other models^33^. Hasan et al. chose the optimal polyp detection strategy by combining different CNN architectures and feature extractors^34^. In addition, Tang et al. utilized transfer learning for computer-aided colonoscopy polyp detection. By applying a pre-trained CNN model to a colonoscopy image dataset, the method can effectively identify and detect polyps in the colon. Compared to training the model from scratch, it has faster training convergence and better generalization ability^35^.

Although the two-stage polyp detection model is the mainstream target detection method, the high time complexity prevents its practical application in medicine. For example: when the R-CNN model extracts the candidate frame of the polyp image, about 2000 candidate areas/candidate frames are generated, and each candidate area/candidate frame must enter the CNN network for feature extraction and SVM classification. This creates a lot of redundant operations, and thus takes a lot of training time. In addition, because the fully connected layer in the CNN network needs to input a fixed-size picture, and the preprocessing operations such as cropping and correcting the picture after generating the candidate area will affect the quality and content of the picture.

### Single-stage polyp detection model

Liew et al. designed a new network, ResNet-50, based on a residual network and incorporating principal component analysis and AdaBoost integrated learning. The method merges three publicly available datasets, CVC-ClinicDB, STIS-LaribPolypDB, and Kvasir, for training the designed model and applies techniques such as contrast enhancement, image thresholding, and median filtering to it to reduce the interference of noise^36^. Influenced by the DETR detection model, Shen et al. designed an end-to-end Convolutional Transformer Network (COTR). This network takes into account the slower convergence rate of DETR and embeds a convolutional layer into the Transformer encoder for accelerating model convergence and feature reconstruction^37^. Wang et al. designed new lightweight models VGGNets-GAP and ResNtes-GAP by introducing global average pooling based on two networks, VGGNets, and ResNtes, from the perspective of improving the accuracy and reducing the model complexity^38^. Qadir et al. introduced a two-dimensional Gaussian mask in a single feedforward network model to reduce the leakage rate of polyps. Experiments show that this method performs outstandingly in the case where the polyp boundary is more ambiguous with the background^39^. Facing the challenges posed by heterogeneous polyp datasets, Li et al. proposed a low-rank module to achieve accurate segmentation of polyps to enhance the generalization ability of the model, using the high-resolution HRNet model as a benchmark^40^. Nisha et al. proposed a dual-path CNN to detect the presence of polyps in colonoscopy images. Compared with traditional deep learning methods, this method learns fewer parameters and reduces computational complexity^41^.

You Only Look Once (YOLO) series models are the more mainstream target detection algorithm nowadays, based on which some scholars have proposed an improved version of YOLO to achieve more accurate recognition^42^. Based on the YOLO network framework, Luo et al. designed a polyp detection system to meet the needs of the clinical environment, which has a high PDR, and the effect is especially obvious on small polyps^43^. Guo et al. designed a new polyp detection algorithm based on YOLOv3 combined with an active learning approach from the perspective of reducing the detection time, which can reduce the false-positive rate in the automatic detection of colon polyps^44^. Cao et al. investigated the difficulty of detecting small gastric polyps for different sizes of gastric polyps, with a special focus on small gastric polyps, and constructed a new feature extraction module and feature fusion module in the YOLOv3 model. This method can combine the semantic information of high-level feature maps with low-level feature maps, which is effective for the detection of small gastric polyps^45^. Pacal et al. applied a cross-stage partial network to the entire architecture and redesigned the backbone structure to address the shortcomings of YOLOv4. The real-time performance and accuracy of this approach far exceeded that of the initial model on the publicly available dataset^46^. Chou et al. first applied discrete wavelet transform to extract the texture features of polyps to enhance the unobvious texture features in polyp images, and then used the pattern-based generative adversarial network to enhance the image data, and finally detected polyps based on YOLOv4. Polyps are better detected^47^.

Although the single-stage polyp detection model has a certain advantage in time, there are many polypoid structures with strong edges in the colon, including colonic folds, blood vessels, mirror lights, lumen areas, air bubbles, etc. These complex colonic environments lead to Too many false positives appear in the detection effect of the algorithm. Moreover, some algorithms are not effective in detecting the presence of multiple polyps or small polyps in a picture.

## Methods

### Architecture of the proposed network

**Figure 1:**
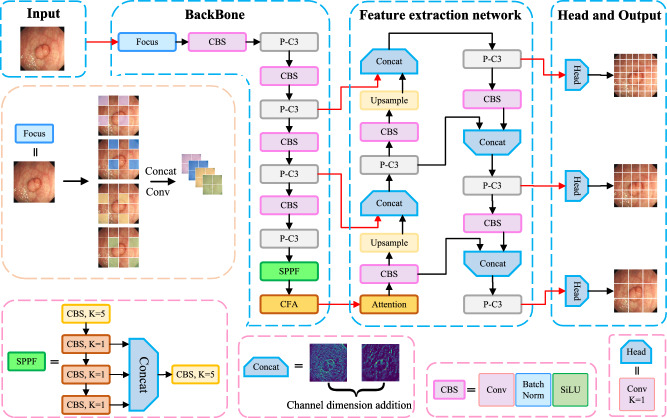
Overall architecture of the proposed scheme.

In the case of limited data sets, in order to reduce the missed or false detection rate of polyps, this paper proposes a multi-attention mechanism colorectal cancer polyp detection model based on YOLOv5. The network structure of the model consists of image input, backbone, neck and prediction head, and its structure is shown in Fig. 1.

In Fig. 1 the *K* represents the size of the convolution kernel. For example, when *K* is 5, it means that the size of the convolution kernel is $5\times 5$.

In this model, New CSP-DarkNet53 as its backbone network uses Focus, C3, Spatial Pyramid Pooling-Fast (SPPF) and Context Feature Augmentation (CFA) with convolution-batch normalization-ReLU activation (CBL) as the basic convolution unit to extract features from colorectal images. The Focus operation achieves high-quality downsampling, which splits a high-resolution feature map into multiple low-resolution feature maps using a slice operation. SPPF is a spatial pyramid pooling layer, which is designed to further increase the receptive field of the feature map. It enables polyps to be well detected when images are input at different scales and effectively avoids the image distortion problem caused by cropping and scaling operations on colorectal images.

SPPF, which stacks 3 identical max-pooling layers with convolution kernel size 5 $\times$ 5 in series, further increases the receptive field through continuous maximum pooling, and solves the problem of repeated extraction of polyp feature information by the neural network. However, this direct fusion of information of different densities will lead to semantic conflicts, limit the expression of multi-scale features, and easily make micro-polyp features submerged in conflicting information. In order to enable micropolyps to be detected, a CFA is designed in this paper, which uses expanded convolution to extract contextual information in different receptive fields to enhance feature expression capabilities and integrate it on top of the backbone network. A coordinated attention mechanism is connected to the path aggregation network (PAN) after CFA, the purpose of which is to enhance the channel connection between each feature, improve the detection accuracy, and ensure the running speed at the same time.

The neck network of the model uses the PAN structure to fuse the feature information of polyps. The PAN introduces bottom-up pathways to gradually aggregate and integrate the polyp features of different scales, thus enabling the network to provide a more comprehensive and rich representation of the polyp features. First, the network performs upsampling from top to bottom, so that the underlying feature map contains more semantic information of the image, and secondly, it performs downsampling from bottom to top, so that the top layer structure of the network can express more accurate location information of polyps. Finally, the two features are fused, so that the polyp feature information and location information can be reflected in the feature maps of each size to ensure an accurate prediction of polyps. To ensure that the model extracts the feature information of polyps more accurately, this paper introduces coordinated attention into the PAN structure. Finally, the feature information of three different scales is output as the prediction head to detect polyps of different scales.

### Input

**Figure 2:**
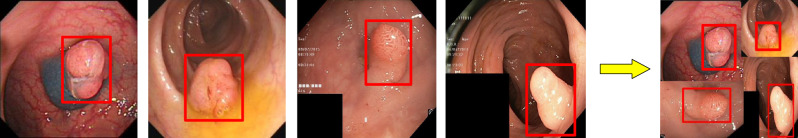
Mosaic data enhancement diagram. The red squares in the figure are the bounding boxes of the polyps.

In the process of polyp image preprocessing, due to the lack of data volume, it takes a lot of manpower and time to label the data at the same time, and the target detection algorithm needs a large amount of high-quality data for model training. Therefore, in the input stage, this paper first adaptively scales the input image, and uses the Kmeans++ clustering algorithm to automatically learn and adjust the size of the anchor box to achieve better prediction of the target location of colorectal polyps. On this basis, the Mosaic data augmentation method is used to address the lack of data. This data enhancement method randomly selects four images from the data set, and combines the rotated, scaled, and deformed four images to form a new polyp image. The basic principle of Mosaic data enhancement is shown in Fig. 2 below.

### P-C3

**Figure 3:**
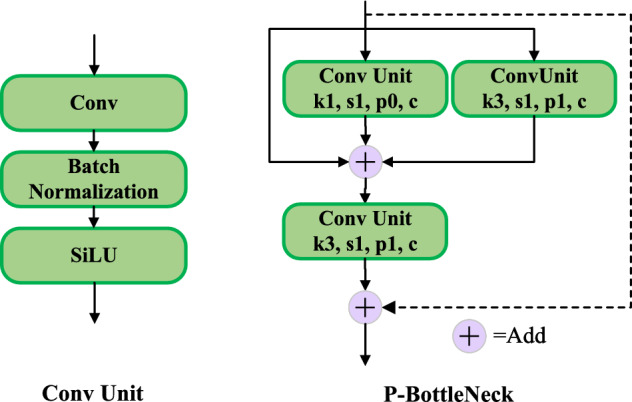
The structure of P-BottleNeck.

To further improve the feature representation of the model, a new structure called P-BottleNeck is designed in this paper. The details of P-BottleNeck is shown in Fig. 3. The two Convs units are connected in parallel with an extra shortcut to prevent the loss of polyp features. Then an add operation is performed on a selectable residual link after a 3$\times$3 convolution. where, *k*1 and *k*3 represent convolution kernels of sizes $1\times 1$ and $3\times 3$, respectively, *s*1 represents a convolution with a step size of 1, *p*0 represents a padding of 0 in the convolution, and *c* represents the channel of the convolution.

**Figure 4:**
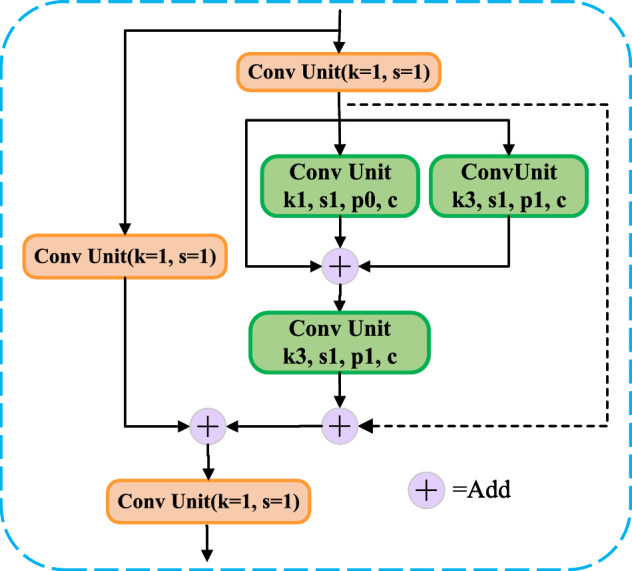
The structure of P-C3.

A new type of cross-stage partial network is designed using the P-BottleNeck structure and named P-C3. The details of the P-C3 module is shown in Fig. 4, where the input features undergo a layer of convolution into the P-BottleNeck structure, connected with an additional convolution to achieve a richer combination of gradients. Finally, the output features of the module are obtained after a 1$\times$1 convolution.

We introduce the P-C3 module into the backbone and neck of the model. In the backbone network, we use the P-C3 module with residual structure, which enhances the feature extraction and mitigates the problem of gradient disappearance. The P-C3 structure deepens the depth of the network and enlarges the receptive field, which enables the model to extract richer polyp feature informations and enhances the feature expression ability.

### CFA module

During a colonoscopy, polyps are difficult to detect due to their small size. The limitations of the network and the imbalance of the training dataset are the main reasons for the poor performance of tiny object detection. Therefore, this paper designs a contextual feature fusion module, which uses dilated convolution to extract the contextual information of different receptive fields, and fuses it to the top of the backbone network to enhance the contextual feature information of tiny polyps.

**Figure 5:**
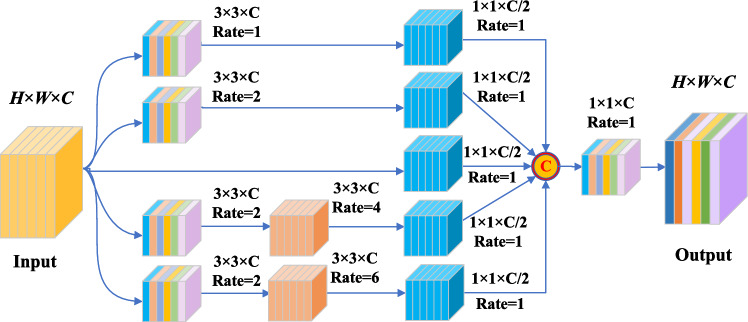
The structure of context feature augmentation.

In this paper, dilated convolutions with different dilated convolution rates are used to obtain contextual information of different receptive fields to enrich the contextual information of PAN, and its structure is shown in Fig. 5.

The CFA module includes four parallel context reasoning branches, aiming to leverage contexts of different sizes for decentralized discovery. The first branch contains one 3$\times$3 dilated convolution with a dilation rate 1, and the second branch contains one 3$\times$3 dilated convolution with a dilation rate 2. The role of these two branches is to be used to access the local context information. The third branch sequentially stacks two 3$\times$3 dilated convolutions with dilation rates 2 and 4, and the fourth branch sequentially stacks two 3$\times$3 dilated convolutions with dilation rates 3 and 6, which are used to access larger contexts with larger dilation rates. Then, each branch reduces the channel by a 1$\times$1 convolution with a dilation rate 1, and the reduced four feature maps are spliced in the channel dimension. Finally, the spliced feature maps are again fused with polyp features from different receptive fields using one 1$\times$1 dilated convolution with a dilation rate 1 to output the final context feature-enhanced map.

### Coordinate attention mechanism

The attention mechanism enables the model to better focus on polyp feature information and suppress non-critical feature information with low weight, enabling the model to extract more accurate semantic information about polyps. Currently, the mainstream attention mechanisms contain Squeeze-and-Excitation attention (SE), Convolutional Block Attention Module (CBAM), etc. The SE enhances the critical information in the feature map by learning the importance of global channels. However, the SE only considers the encoding of inter-channel information and ignores the importance of polyp location information. The CBAM solves the shortcomings of SE by combining channel attention and spatial attention and learns the importance of each spatial location through the spatial attention mechanism. However, its high computational complexity makes it difficult to apply to real-time detection of polyps.

**Figure 6:**
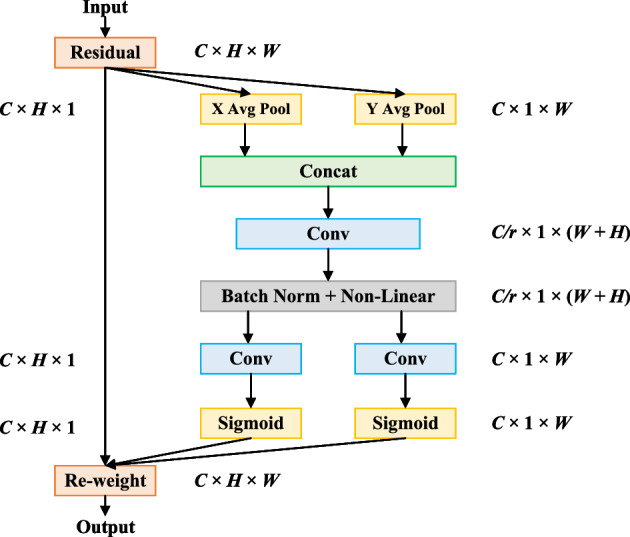
The coordinate attention mechanism.

In the process of algorithm design, to make the model locate and identify polyps more accurately, and to improve the polyp detection accuracy under the premise of ensuring the inference speed, we introduce a simple and flexible coordinated attention mechanism (CAM) to pay special attention to the important regions of the image. The specific process of this attention is shown in Fig. 6.

The CAM not only captures the information of polyp features across channels and enhances the channel connection among features, but also captures the information of direction perception and position perception, which helps the model to accurately detect polyps and achieve precise localization. In addition, The CAM attention is flexible and lightweight and can be applied to real-time polyp detection tasks.

In order to avoid all the spatial information being compressed into the channel, resulting in the inability to capture long-range spatial interaction with precise location information, the coordinated attention mechanism decomposes the global average pooling on the spatial dimension into two directions of height and width, and obtains two scales respectively. The feature maps of *C*×*H*×1 and *C*×1×*W* are as follows:

1 $$\begin{aligned} \begin{aligned} Z_{c}^{h} =\frac{1}{W}{\textstyle \sum _{0\le i\le W}\left| x_{c}\right. \left( h,i\right) }\\ Z_{c}^{w} =\frac{1}{H}{\textstyle \sum _{0\le i\le H}\left| x_{c}\right. \left( j,w\right) } \end{aligned} \end{aligned}$$

where *x* represents the feature map, *h*, *w*, and *c* represent the height, width, and number of channels of the feature map. $Z_{c}^{h}$ and $Z_{c}^{w}$ represent the perceptual attention maps obtained by feature aggregation along the two spatial dimensions of height and width, respectively. The *i* and *j* represent the positional information of the feature maps in terms of height and width.

Next, the feature map *C*×1×*W* with the width dimension of the global perceptual field is obtained by transforming it into *C*×*W*×1 and stitching it with the feature map *C*×*H*×1 on the height, and reducing the channel dimension to 1/*r* of the original by the shared convolution module to obtain the feature map $F_1$. Then, the feature map $F_1$, which is processed by batch normalization, is activated using the Sigmoid activation function to obtain the feature map $f\in R^{C/r\times (H+W)\times 1}$, as follows:

2 $$\begin{aligned} f=\delta \left( F_{1}\left( \left[ Z^{h},Z^{w}\right] \right) \right) \end{aligned}$$

where $Z^{h}$ and $Z^{w}$ represent the feature maps in both height and width dimensions, and $\delta$ represents the sigmoid activation function.

Then, the feature map *f* is restored to the same number of channels along the spatial dimension as the original feature map size to obtain the feature maps $f_{h} \in R^{C/r\times H\times 1}$ and $f_{w} \in R^{C/r\times W\times 1}$. The feature maps $f_{h} \in R^{C/r\times H\times 1}$ and $f_{w} \in R^{C/r\times W\times 1}$ are Sigmoid activated in turn to obtain the attention weights $g^{h}\in R^{C\times H\times 1}$ in height and $g^{w}\in R^{C\times W\times 1}$ in width direction of the original feature map. the equations are shown below:

3 $$\begin{aligned} \begin{aligned} g^{h}=\sigma \left( F_{h}\left( f^{h}\right) \right) \\ g^{w}=\sigma \left( F_{w}\left( f^{w}\right) \right) \end{aligned} \end{aligned}$$

Finally, the attention weights $g^{h}$ and $g^{w}$ in the height and width directions obtained above are weighted and multiplied on the original feature map to output the polyp feature map with attention weights, and the equations are shown below:

4 $$\begin{aligned} y_{c}\left( i,j\right) =x_{c}\left( i,j\right) \times g_{c}^{h}\left( i\right) \times g_{c}^{w}\left( j\right) \end{aligned}$$

### Loss function

The loss function of the polyp detection model used in this paper includes classification loss, regression loss and confidence loss. Its loss function can be described as follows:

5 $$\begin{aligned} Loss=L_{cls} +L_{box} +L_{obj} \end{aligned}$$

where $L_{cls}$ stands for classification loss, $L_{boxes}$ stands for regression loss, and $L_{obj}$ stands for confidence loss. In which the regression loss function of the bounding box is calculated as:

6 $$\begin{aligned} \begin{aligned} L_{box}=\lambda _{coord}\sum _{i=0}^{S^{2}}\sum _{i=0}^{B}I_{i,j}^{obj}\left( 1-CIoU\right) \\ CIoU=IoU-\frac{d^{2}}{c^{2}}-\alpha v,IoU=\frac{\left| B\cap B^{g}\right| }{\left| B\cup B^{g}\right| }\\ \alpha =\frac{v}{\left( 1-IoU\right) +v},v=\frac{4}{\pi ^{2}}\left( tan^{-1}\frac{\omega ^{g}}{h^{g}}-tan^{-1}\frac{\omega }{h}\right) ^{2} \end{aligned} \end{aligned}$$

where $\lambda _{coord}$ represents the regression loss coefficient of the bounding box, $I_{i,j}$ represents whether the *j*th anchor in the *i*-th cell contains the target polyp, *B* represents the prediction box, and $B^{g}$ represents the true box. *c* represents the diagonal length of the smallest rectangle that can contain both the prediction box and the true box enclosed, and *d* represents the Euclidean distance between the centroids of the true and prediction boxes. The parameter $\alpha$ represents the positive weight, *v* measures the consistency of the aspect ratio.

### Ethical statements

We confirm that all methods in this paper were carried out in accordance with relevant guidelines and regulations, and all experimental protocols were approved by Ethics Committee of Huai’an Second People’s Hospital. We confirm that informed consent was obtained from all subjects and/or their legal guardian(s).

## Experiments and results

### Dataset and implementation

**Figure 7:**
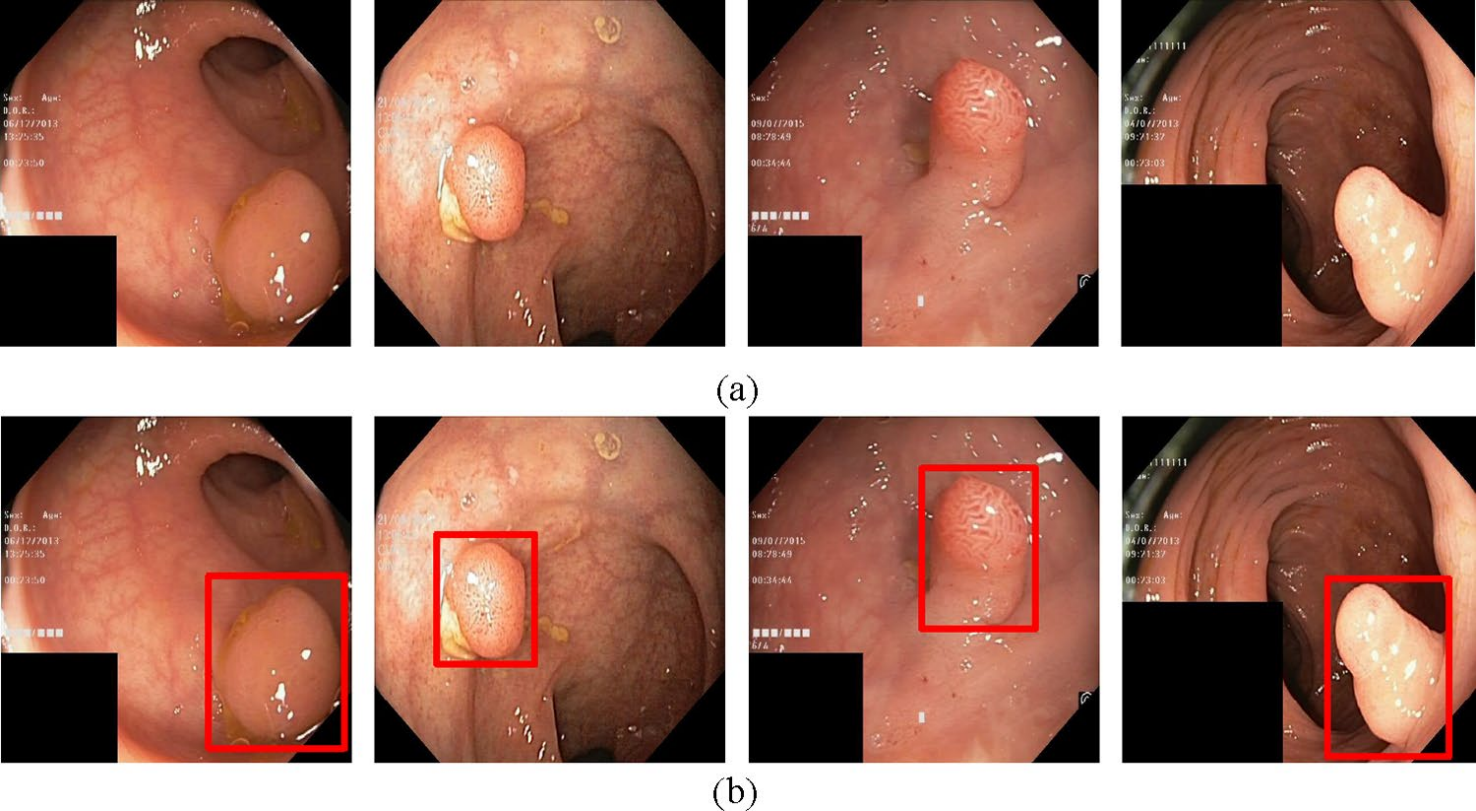
The polyp object detection WCYZ dataset. (**a**) Polyp images samples. (**b**) Polyp images with bounding boxes. The red squares in the figure are the bounding boxes of the polyps.

To validate the superiority of the proposed method in this paper, we collected 1200 colorectal images containing 1–4 colon polyps each from the endoscopy center of a local hospital, constructed a WCYZ dataset, and divided the training set, test set and validation set in the ratio of 8:1:1. Figure 7a shows some example plots of polyp images, and Fig. 7b illustrates the real boxes labeled using bounding boxes. Stochastic gradient descent (SGD) was chosen as the optimizer for all experiments with an initial learning rate of 0.01, a momentum of 0.9, a batch size of 16, and 200 training epochs.

### Evaluation metrics

In this paper, precision, recall and F-score are used to evaluate the detection performance of the model, which are defined as follows:

7 $$\begin{aligned} \begin{aligned} Precision=\frac{TP}{TP+FP} \end{aligned} \end{aligned}$$

8 $$\begin{aligned} \begin{aligned} Recall=\frac{TP}{TP+FN} \end{aligned} \end{aligned}$$

Among them, *TP*, *FP*, and *FN* represent the number of true positives, false positives, and false negatives, respectively, that is, the number of polyps that were correctly detected and labeled, the number of falsely detected polyps, and the number of undetected polyps. *F-score* provides an overall evaluation by comprehensively considering the precision and recall indicators. The specific formula is defined as follows:

9 $$\begin{aligned} \begin{aligned} F-score=2\times \frac{precision\times recall}{precision+ recall} \end{aligned} \end{aligned}$$

### Analysis of polyp detection results

#### Ablation experiments

In order to evaluate the contribution of the introduction of the P-C3 model, contextual feature augmentation and coordinate attention mechanism modules to network detection capabilities, this paper conducts experiments using YOLOv5 as a benchmark. The experimental results on the WCYZ dataset listed in Table 1 shows the performance indicators of the model after adding the P-C3 module, contextual feature augmentation module, and the attention mechanism.

**Table 1:** Results of ablation experiments on the WCYZ.

Group	CFA	CAM	P-C3	Parameters	Precision	Recall	F-score	Time (s)
1				7055192	0.871	0.877	0.874	**0.025**
2	$\checkmark$			21520118	0.874	0.902	0.888	0.029
3	$\checkmark$	$\checkmark$		21697294	0.882	0.911	0.896	0.031
4	$\checkmark$	$\checkmark$	$\checkmark$	22933134	**0.916**	**0.923**	**0.919**	0.034

In the table, the optimal results obtained by each index are in bold.

As can be seen from Table 1, the introduction of the P-C3 module allows the network to extract richer polyp features and achieve higher detection accuracy. The CFA module has made a huge contribution to the improvement of the detection rate. This is because there are polyps of different sizes in the data set. The CFA module uses expanded convolution to extract the context information of different receptive fields, and integrates it into the PAN to improve the context feature information of tiny polyps, enabling the model to perform better in the face of small target polyps, thereby improving the recall of the model.

#### Visualization result analysis

**Figure 8:**
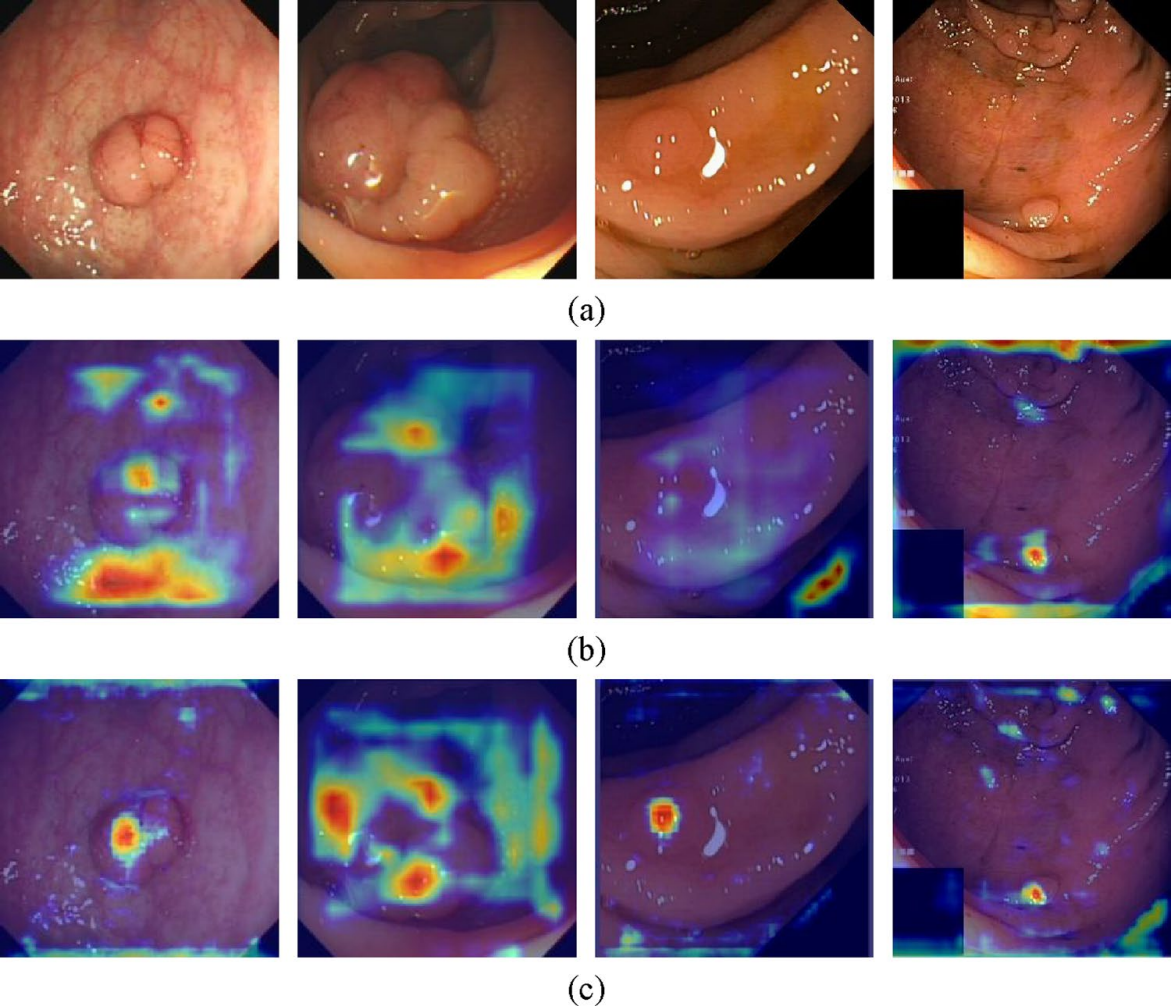
Heat maps of the polyp in the dataset. (**a**) Original image of polyps. (**b**) Heat map of the polyp in YOLOv5. (**c**) Heat map of the polyp in the improved model. (The heat map was generated using grad-cam 1.4.6. URL=https://github.com/jacobgil/pytorch-grad-cam).

In this paper, heat maps were used to visualize the results of the polyp detection. By observing the heat distribution in the heat map, the model’s ability to detect polyps and the accuracy of polyp localization can be visually assessed. The details of the visualization are shown in Fig. 8.

Comparing the polyp heat maps of the original model in Fig. 8b and the improved model in Fig. 8c, it can be seen that the improved network model has gained an improvement in the accuracy and coverage of focusing on the target region of polyp detection, which proves that our method can help the deep convolutional network to extract more critical polyp feature information.

#### Comparison of experimental results with other methods

In the field of colorectal cancer research, we should usually adjust the parameters or rule codes of the algorithm model to ensure the priority of the recall rate, find out more polyps that may have abnormalities, and reduce the risk of “missed detection”. Next, in order to verify the effectiveness of the proposed method, we used popular deep learning-based detection algorithms R-CNN^48^, Faster R-CNN^49^, YOLOv4^50^, YOLOv7^51^, YOLOv8^52^ and RT-DETR-R50^53^ to conduct comparative experiments on model detection accuracy and speed. The quantitative results of each detection model on our test set are shown in Table 2.

**Table 2:** Comparison of different methods on the WCYZ.

Methods	Precision	Recall	F-score	Time (s)
R-CNN	0.907	0.889	0.897	1.180
Faster R-CNN	0.914	0.896	0.905	0.375
YOLOv4	0.881	0.879	0.880	0.033
YOLOv7	0.783	0.764	0.773	0.024
YOLOv8	0.904	0.878	0.891	**0.020**
RT-DETR-R50	0.868	0.872	0.870	0.021
Ours	**0.916**	**0.923**	**0.919**	0.034

In the table, the optimal results obtained by each index are in bold.

It can be seen from the comparative data in Table 2 that our method shows obvious advantages in the detection rate and speed of polyps. Compared with the two-stage R-CNN model, recall has increased by 2.7 percentage points, and the speed has been greatly reduced. Compared with the single-stage YOLOv8 detection algorithm, recall has increased by 4.5 percentage points, reaching 92.3%.

#### Test results visualization

**Figure 9:**
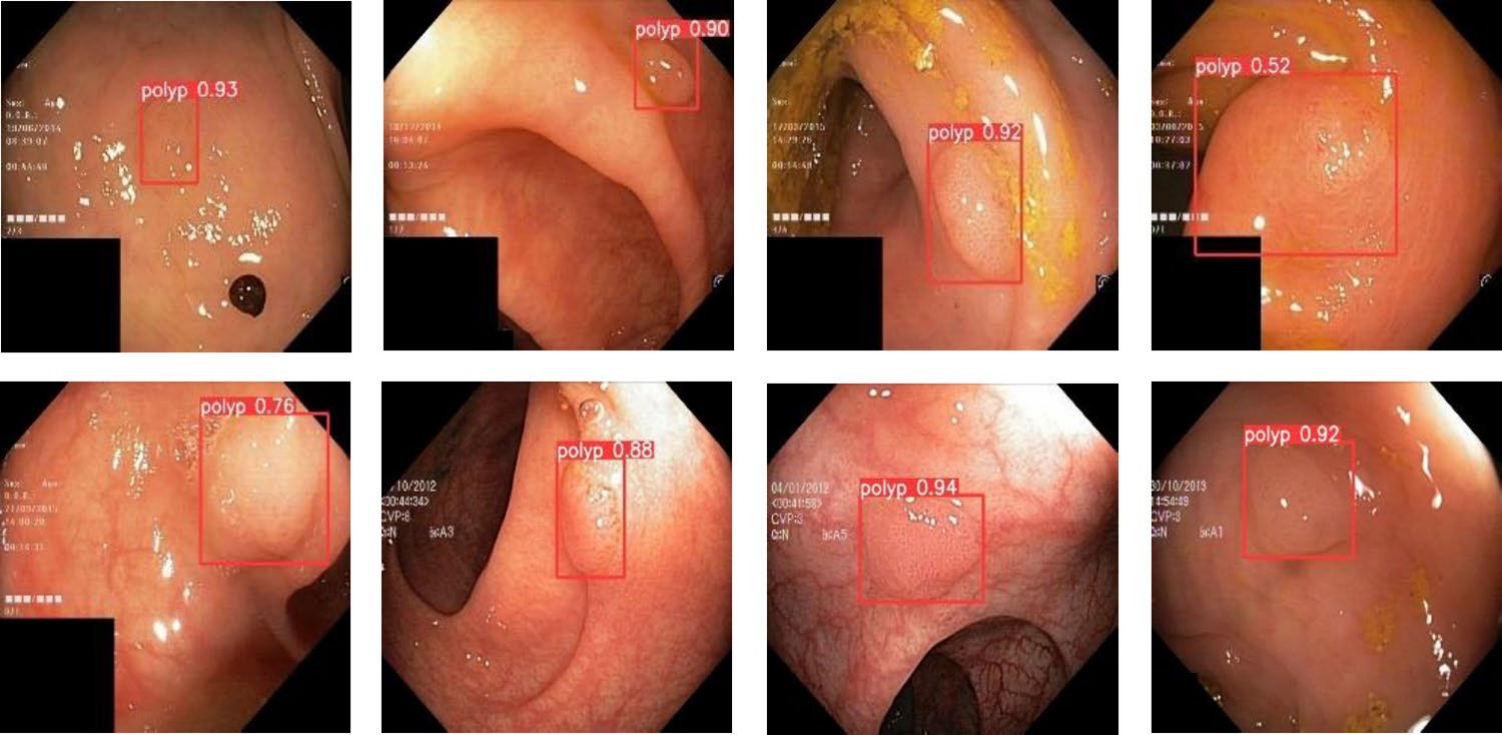
A subset of the detection results of polyps showing low contrasts to the background in WCYZ data set. The red squares in the figure are the bounding boxes of the polyps.

**Figure 10:**
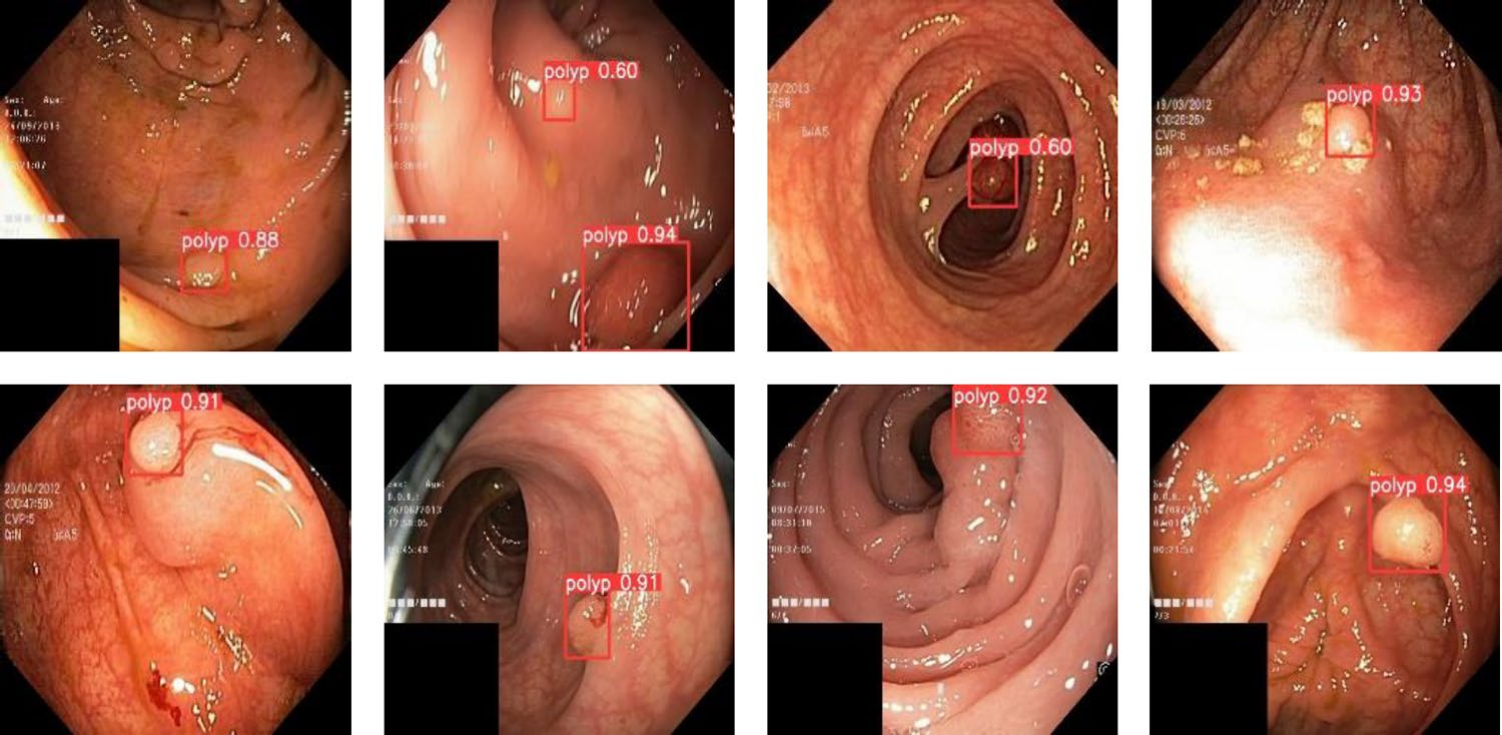
A subset of the detection results of small target polyps in WCYZ data set. The red squares in the figure are the bounding boxes of the polyps.

**Figure 11:**
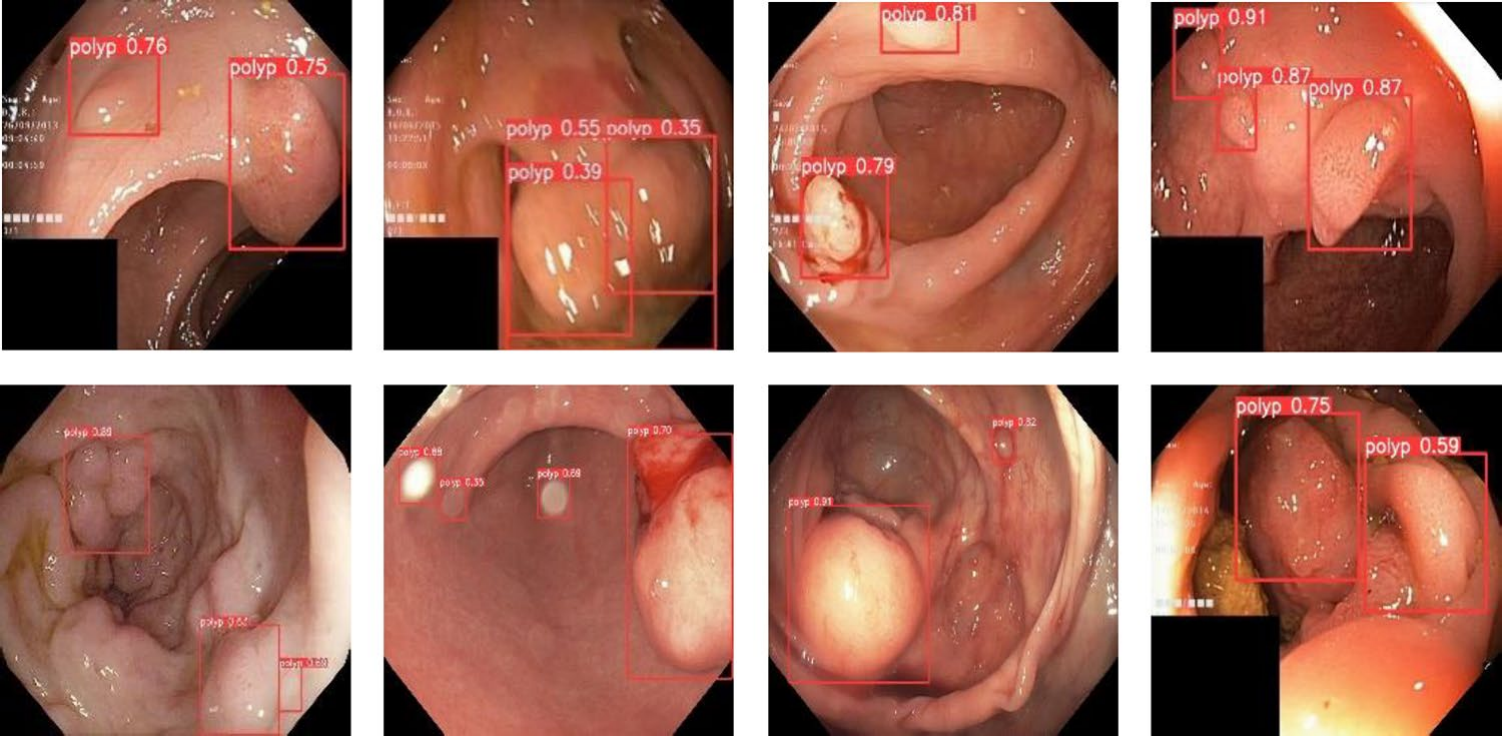
A subset of the detection results of multiple target polyps in WCYZ data set. The red squares in the figure are the bounding boxes of the polyps.

To visualize the test results of polyp detection, Figs. 9, 10 and 11 show the detection results of some polyps in the test set.

In the process of polyp detection, the polyps in the image have a similar color to the background, making their detection difficult and preventing accurate identification. For processing images with weak contrast between polyps and background, the polyp detection results are shown in Fig. 9. From the figure, we can see that our model has obvious effect for processing polyps with weak contrast.

In the colon images, smaller polyps are difficult to be detected. The experimental results of the improved model for smaller polyps detection are shown in Fig. 10. We can see that, from the figure, the detection algorithm in this paper can accurately identify and localize them. This is due to the introduction of CFA, which enables the model to handle tiny polyps well.

In addition, when detecting polyps, there may be multiple polyps, and the method proposed in this paper can also handle this situation well. The polyp detection results are shown in Fig. 11. We can see that, from the figure, the method we proposed shows good performance for the detection of multiple polyps of different sizes.

#### Comparison of polyp detection results

**Figure 12:**
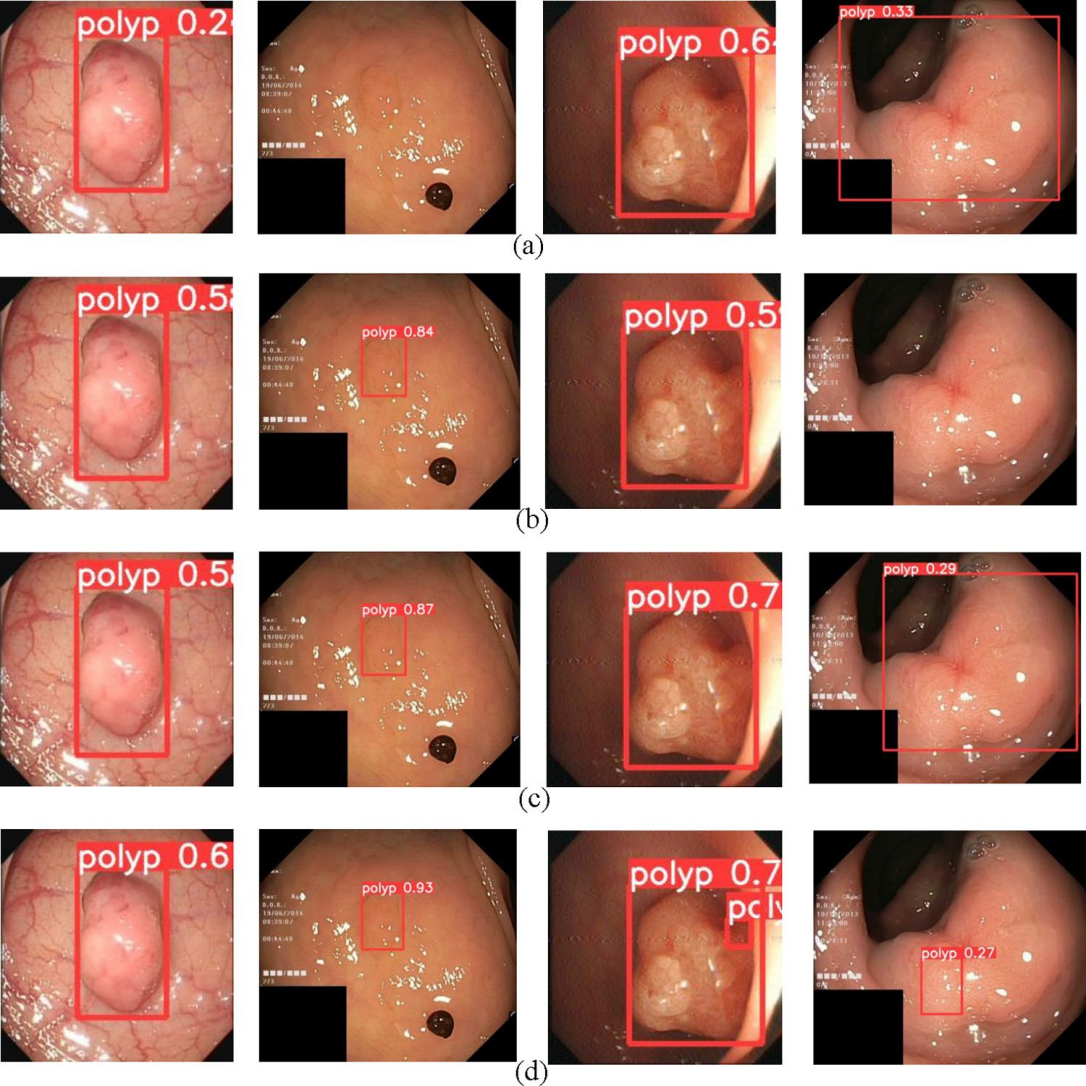
Comparison of the improved model with the original. (**a**) Detection results of YOLOv5. (**b**) Detection results of YOLOv7. (**c**) Detection results of YOLOv8. (**d**) Detection results of Ours.

In order to verify the effectiveness of our model in real detection, we compare the detection results with those of the latest YOLOv5, YOLOv7 and YOLOv8 modeling algorithms. We used four sets of scene images to qualitatively evaluate the detection performance of the model, and the polyp detection results are shown in Fig. 12.

In the first set of images, YOLOv5 faced challenges in accuracy, YOLOv7 and YOLOv8 had more similar performance, and our model performed well. In the second set of images, due to the weak contrast between the polyps and the image background, the YOLOv5 network model has missed detection, while the proposed model can accurately detect polyps with an accuracy of 93%, which proves that the improved model can efficiently deal with the targets with weak contrast. The YOLOv7 and YOLOv8 algorithms perform relatively better, but still lower than our proposed model. In the third set of images, due to the presence of tiny polyps, the YOLOv5, YOLOv7, and YOLOv8 models were not effective in detecting tiny polyps. In contrast, the improved model was able to accurately capture the tiny polyp feature information and achieve precise localization. In the fourth set of images, the YOLOv7 model fails to detect polyps, while the YOLOv5 and YOLOv8 models localize polyps inaccurately, in contrast to our method, which accurately localizes polyp location information.

Overall, our improved model can achieve excellent performance in the polyp detection process.

## Conclusion

The detection of polyps in colonoscopy images is an important part of medical image recognition. Due to the weak contrast of polyps in colonoscopy images and the small size of some polyps, it is difficult to detect polyps. This paper proposes a more refined polyp detection method on the basic network of YOLOv5, aiming to improve the detection rate of polyp detection and avoid missed detection. In this method, a new type of module named P-C3 is proposed and added to the backbone and neck network. The CFA is introduced at the bottom of the DarkNet53 backbone network to improve the expressiveness of the model. On this basis, the output features are passed into the CA module to make the model pay more attention to the polyp features. This study will contribute to the field of colon cancer polyp detection, which can greatly reduce the misdiagnosis rate of clinicians in endoscopic diagnosis and treatment, and will be beneficial to physicians in their clinical work.

## Data Availability

The dataset analysed during the current study is available from the corresponding author on reasonable request.

## References

[1] Cao W, Chen H-D, Yu Y-W, Li N, Chen W-Q (2021). Changing profiles of cancer burden worldwide and in china: A secondary analysis of the global cancer statistics 2020. Chin. Med. J..

[2] Ichkhanian Y, Zuchelli T, Watson A, Piraka C (2021). Evolving management of colorectal polyps. Ther. Adv. Gastrointest. Endosc..

[3] Kudo S-E (2019). Artificial intelligence and colonoscopy: Current status and future perspectives. Dig. Endosc..

[4] Amini M, Rezasoltani S, Pourhoseingholi MA, Asadzadeh Aghdaei H, Zali MR (2022). Evaluating the predictive performance of gut microbiota for the early-stage colorectal cancer. BMC Gastroenterol..

[5] Maida M (2019). Quality measures improving endoscopic screening of colorectal cancer: A review of the literature. Expert Rev. Anticancer Ther..

[6] Tavanapong W (2022). Artificial intelligence for colonoscopy: Past, present, and future. IEEE J. Biomed. Health Inform..

[7] Lima ACDM (2023). A two-stage method for polyp detection in colonoscopy images based on saliency object extraction and transformers. IEEE Access.

[8] Lalinia M, Sahafi A (2024). Colorectal polyp detection in colonoscopy images using yolo-v8 network. Signal Image Video Process..

[9] Doubeni CA (2018). Effectiveness of screening colonoscopy in reducing the risk of death from right and left colon cancer: A large community-based study. Gut.

[10] Waldmann E (2021). Association of adenoma detection rate and adenoma characteristics with colorectal cancer mortality after screening colonoscopy. Clin. Gastroenterol. Hepatol..

[11] Zhang J (2020). Colonoscopic screening is associated with reduced colorectal cancer incidence and mortality: A systematic review and meta-analysis. J. Cancer.

[12] Mahmud N, Cohen J, Tsourides K, Berzin TM (2015). Computer vision and augmented reality in gastrointestinal endoscopy. Gastroenterol. Rep..

[13] Ng S, Sreenivasan AK, Pecoriello J, Liang PS (2020). Polyp detection rate correlates strongly with adenoma detection rate in trainee endoscopists. Dig. Dis. Sci..

[14] Barua I (2021). Artificial intelligence for polyp detection during colonoscopy: A systematic review and meta-analysis. Endoscopy.

[15] Hoerter N, Gross SA, Liang PS (2020). Artificial intelligence and polyp detection. Curr. Treat. Options Gastroenterol..

[16] Sinonquel P (2021). Real-time unblinding for validation of a new cade tool for colorectal polyp detection. Gut.

[17] Soons E (2022). Real-time colorectal polyp detection using a novel computer-aided detection system (cade): A feasibility study. Int. J. Colorectal Dis..

[18] Krishnan, S., Yang, X., Chan, K., Kumar, S. & Goh, P. Intestinal abnormality detection from endoscopic images. in *Proceedings of the 20th Annual International Conference of the IEEE Engineering in Medicine and Biology Society. Vol. 20 Biomedical Engineering Towards the Year 2000 and Beyond (Cat. No. 98CH36286)*, vol. 2, 895–898 (IEEE, 1998).

[19] Kang, J. & Doraiswami, R. Real-time image processing system for endoscopic applications. in *CCECE 2003-Canadian Conference on Electrical and Computer Engineering. Toward a Caring and Humane Technology (Cat. No. 03CH37436)*, vol. 3, 1469–1472 (IEEE, 2003).

[20] Bernal J (2015). Wm-dova maps for accurate polyp highlighting in colonoscopy: Validation vs. saliency maps from physicians. Comput. Med. Imaging Graph..

[21] Alexandre, L. A., Casteleiro, J. & Nobreinst, N. Polyp detection in endoscopic video using svms. in *Knowledge Discovery in Databases: PKDD 2007: 11th European Conference on Principles and Practice of Knowledge Discovery in Databases, Warsaw, Poland, September 17-21, 2007. Proceedings 11*, 358–365 (Springer, 2007).

[22] Li, P., Chan, K. L. & Krishnan, S. M. Learning a multi-size patch-based hybrid kernel machine ensemble for abnormal region detection in colonoscopic images. in *2005 IEEE Computer Society Conference on Computer Vision and Pattern Recognition (CVPR’05)*, vol. 2, 670–675 (IEEE, 2005).

[23] Haj-Manouchehri A, Mohammadi HM (2020). Polyp detection using CNNs in colonoscopy video. IET Comput. Vis..

[24] Mostafiz R, Hasan M, Hossain I, Rahman MM (2020). An intelligent system for gastrointestinal polyp detection in endoscopic video using fusion of bidimensional empirical mode decomposition and convolutional neural network features. Int. J. Imaging Syst. Technol..

[25] Billah M, Waheed S, Rahman MM (2017). An automatic gastrointestinal polyp detection system in video endoscopy using fusion of color wavelet and convolutional neural network features. Int. J. Biomed. Imaging.

[26] Hasan MM, Islam N, Rahman MM (2022). Gastrointestinal polyp detection through a fusion of contourlet transform and neural features. J. King Saud Univ. Comput. Inf. Sci..

[27] Bernal J (2017). Comparative validation of polyp detection methods in video colonoscopy: Results from the MICCAI 2015 endoscopic vision challenge. IEEE Trans. Med. Imaging.

[28] Tajbakhsh, N., Gurudu, S. R. & Liang, J. Automatic polyp detection in colonoscopy videos using an ensemble of convolutional neural networks. in *2015 IEEE 12th International Symposium on Biomedical Imaging (ISBI)*, 79–83 (IEEE, 2015).

[29] Qadir HA (2019). Improving automatic polyp detection using CNN by exploiting temporal dependency in colonoscopy video. IEEE J. Biomed. Health Inform..

[30] Mo, X., Tao, K., Wang, Q. & Wang, G. An efficient approach for polyps detection in endoscopic videos based on faster r-CNN. in *2018 24th International Conference on Pattern Recognition (ICPR)*, 3929–3934 (IEEE, 2018).

[31] Qadir, H. A. *et al.* Polyp detection and segmentation using mask r-CNN: Does a deeper feature extractor cnn always perform better? in *2019 13th International Symposium on Medical Information and Communication Technology (ISMICT)*, 1–6 (IEEE, 2019).

[32] Tashk, A. & Nadimi, E. An innovative polyp detection method from colon capsule endoscopy images based on a novel combination of rcnn and drlse. in *2020 IEEE Congress on Evolutionary Computation (CEC)*, 1–6 (IEEE, 2020).

[33] Patel K (2020). A comparative study on polyp classification using convolutional neural networks. PloS One.

[34] Hasan MM, Hossain MM, Mia S, Ahammad MS, Rahman MM (2022). A combined approach of non-subsampled contourlet transform and convolutional neural network to detect gastrointestinal polyp. Multimed. Tools Appl..

[35] Tang C-P, Chen K-H, Lin T-L (2021). Computer-aided colon polyp detection on high resolution colonoscopy using transfer learning techniques. Sensors.

[36] Liew WS, Tang TB, Lin C-H, Lu C-K (2021). Automatic colonic polyp detection using integration of modified deep residual convolutional neural network and ensemble learning approaches. Comput. Methods Programs Biomed..

[37] Shen, Z., Fu, R., Lin, C. & Zheng, S. Cotr: Convolution in transformer network for end to end polyp detection. in *2021 7th International Conference on Computer and Communications (ICCC)*, 1757–1761 (IEEE, 2021).

[38] Wang W (2020). An improved deep learning approach and its applications on colonic polyp images detection. BMC Med. Imaging.

[39] Qadir HA (2021). Toward real-time polyp detection using fully CNNs for 2d gaussian shapes prediction. Med. Image Anal..

[40] Wuyang, L. *et al.* Joint polyp detection and segmentation with heterogeneous endoscopic data. in *3rd International Workshop and Challenge on Computer Vision in Endoscopy (EndoCV 2021): co-located with with the 17th IEEE International Symposium on Biomedical Imaging (ISBI 2021)*, 69–79 (CEUR-WS Team, 2021).

[41] Nisha J, Gopi VP, Palanisamy P (2022). Automated colorectal polyp detection based on image enhancement and dual-path CNN architecture. Biomed. Signal Process. Control.

[42] Wang, C.-Y., Yeh, I.-H. & Liao, H.-Y. M. Yolov9: Learning what you want to learn using programmable gradient information. arXiv preprint arXiv:2402.13616 (2024).

[43] Luo Y (2021). Artificial intelligence-assisted colonoscopy for detection of colon polyps: A prospective, randomized cohort study. J. Gastrointest. Surg..

[44] Guo, Z. *et al.* Reduce false-positive rate by active learning for automatic polyp detection in colonoscopy videos. in *2020 IEEE 17th International Symposium on Biomedical Imaging (ISBI)*, 1655–1658 (IEEE, 2020).

[45] Cao C (2021). Gastric polyp detection in gastroscopic images using deep neural network. PloS one.

[46] Pacal I, Karaboga D (2021). A robust real-time deep learning based automatic polyp detection system. Comput. Biol. Med..

[47] Chou Y-C, Chen C-C (2022). Improving deep learning-based polyp detection using feature extraction and data augmentation. Multimed. Tools Appl..

[48] Girshick, R., Donahue, J., Darrell, T. & Malik, J. Rich feature hierarchies for accurate object detection and semantic segmentation. in *Proceedings of the IEEE Conference on Computer Vision and Pattern Recognition*, 580–587 (2014).

[49] Ren, S., He, K., Girshick, R. & Sun, J. Faster r-cnn: Towards real-time object detection with region proposal networks. *Advances in neural information processing systems* **28** (2015). 10.1109/TPAMI.2016.2577031 27295650

[50] Bochkovskiy, A., Wang, C.-Y. & Liao, H.-Y. M. Yolov4: Optimal speed and accuracy of object detection. arXiv preprint arXiv:2004.10934 (2020).

[51] Wang, C.-Y., Bochkovskiy, A. & Liao, H.-Y. M. Yolov7: Trainable bag-of-freebies sets new state-of-the-art for real-time object detectors. in *Proceedings of the IEEE/CVF Conference on Computer Vision and Pattern Recognition*, 7464–7475 (2023).

[52] Jocher, G., Chaurasia, A. & Qiu, J. Ultralytics YOLO (2023).

[53] Zhao, Y. *et al.* Detrs beat yolos on real-time object detection. arXiv preprint arXiv:2304.08069 (2023).

